# A240 "MY FEELINGS AND MY THOUGHTS ARE MY LIVED EXPERIENCE, NOT THE NUMBERS THEY SHOW ME ON A PIECE OF PAPER”: INDIGENOUS EXPERIENCES OF LIVER TRANSPLANTATION IN BRITISH COLUMBIA, CANADA

**DOI:** 10.1093/jcag/gwac036.240

**Published:** 2023-03-07

**Authors:** C Cassidy-Matthews, M Pearce, T Hussaini, P Spittal, N Caron, C Daley, E Yoshida

**Affiliations:** University of British Columbia, Vancouver, Canada

## Abstract

**Background:**

Indigenous peoples in Canada face inequities in healthcare access due to historic and ongoing colonial systems and structures of oppression. Indigenous peoples face greater health challenges including acute/chronic disease and may require consideration for transplantation including liver, kidney, heart, and lung. In a renal transplant study, Indigenous patients were less likely to undergo transplantation compared to non-Indigenous patients on dialysis; the reasons for this disparity are not known. The experiences of Indigenous patients during the liver transplant process in British Columbia, and how transplant professionals perceive challenges faced by Indigenous people, has not been studied.

**Purpose:**

The purpose of this study is to explore experiences of Indigenous patients during the liver transplant process in British Columbia, and how transplant professionals perceive challenges faced by Indigenous people. This work will provide foundational evidence for improving Indigenous patient care in the transplant process in British Columbia, in response to recommendations made to address systemic and interpersonal racism documented in the In Plain Sight report in 2020.

**Method:**

Thirteen semi-structured qualitative interviews were conducted with Indigenous patients (n=7) and transplant care providers (n=6) across British Columbia, Canada. Interpretive description identified themes to inform clinical approaches and transplant care planning. Themes and related recommendations were validated by Indigenous health experts prior to contextualization.

**Result(s):**

Interviews were conducted via Zoom from April 2021-May 2022. Among liver transplant patient participants: transplants had occurred between 1992-2020; all were women; and the median age at the time of their interview was 58 years. Among transplant care provider participants, roles included nursing, social work, and surgery; 83% were women; and the median number of years in transplant care was ten. Three broad themes were identified: 1) Indigenous transplant patient participants had strong familial, cultural, and financial supports that were essential to accessing liver transplantation; 2) colonialism has created and perpetuated structural barriers preventing many more Indigenous peoples from being considered eligible for liver transplant; 3) anti-Indigenous racism and a lack of cultural safety and humility are barriers for Indigenous peoples who require timely care for liver disease and for establishing trusting relationships with care providers.

**Conclusion(s):**

To our knowledge, this study is the first to explore experiences of Indigenous liver transplant recipients and transplant care providers’ understanding of inequities uniquely experienced by Indigenous peoples. Addressing structural barriers to early linkage to care is needed and training for transplant clinicians on Indigenous histories, complex and intergenerational trauma, and cultural safety is strongly recommended.

**Please acknowledge all funding agencies by checking the applicable boxes below:**

None

**Disclosure of Interest:**

None Declared

